# Evaluation of Open Surgical and Endovascular Treatment Options for Visceral Artery Erosions after Pancreatitis and Pancreatic Surgery

**DOI:** 10.3390/curroncol29040201

**Published:** 2022-03-30

**Authors:** Leon Bruder, Larissa Schawe, Bernhard Gebauer, Jan Paul Frese, Maximilian de Bucourt, Katharina Beyer, Johann Pratschke, Andreas Greiner, Safwan Omran

**Affiliations:** 1 Department of Vascular Surgery, Charité—Universitätsmedizin Berlin, Corporate Member of Freie Universität Berlin and Humboldt-Universität zu Berlin, 12203 Berlin, Germany; leon.bruder@charite.de (L.B.); larissa.schawe@charite.de (L.S.); jan-paul-bernhard.frese@charite.de (J.P.F.); andreas.greiner@charite.de (A.G.); 2 Department of Radiology, Campus Virchow-Klinikum, Charité—Universitätsmedizin Berlin, Corporate Member of Freie Universität Berlin and Humboldt-Universität zu Berlin, 13353 Berlin, Germany; bernhard.gebauer@charite.de; 3 Department of Radiology, Campus Benjamin Franklin, Charité—Universitätsmedizin Berlin, Corporate Member of Freie Universität Berlin and Humboldt-Universität zu Berlin, 12203 Berlin, Germany; maximilian.de-bucourt@charite.de; 4 Department of Surgery, Campus Benjamin Franklin, Charité—Universitätsmedizin Berlin, Corporate Member of Freie Universität Berlin and Humboldt-Universität zu Berlin, 12203 Berlin, Germany; katharina.beyer2@charite.de; 5 Department of Surgery, Campus Virchow-Klinikum and Charité Campus Mitte, Charité—Universitätsmedizin Berlin, Augustenburger Platz 1, Corporate Member of Freie Universität Berlin and Humboldt-Universität zu Berlin, 13353 Berlin, Germany; johann.pratschke@charite.de

**Keywords:** visceral artery erosion, pancreatectomy, pancreatitis, extraluminal hemorrhage, coil embolization

## Abstract

Purpose: To report and compare the results of endovascular and open surgical treatment for erosion bleeding of visceral arteries following pancreatitis and pancreatic surgery. Materials and Methods: This retrospective study included 65 consecutive patients (46 males, mean age 63 ± 14 years) presenting with visceral artery erosions between January 2011 and December 2020. Endpoints were technical success, freedom from reintervention, stent-graft-related complications, and 30-day and one-year mortality. Results: The causes of erosion bleeding included complications of surgical treatment for the pancreas and upper gastrointestinal tract (75%), pancreatitis (19%), and spontaneous bleeding (6%). Pancreatectomy was performed in 34 (52%) patients, representing 2% of all pancreatectomy procedures (*n* = 1645) performed in our hospital during the study period. A total of 37 (57%) patients underwent endovascular treatment (EVT), and 28 (43%) patients had open surgery (OS) as a primary treatment. Eight of 37 (22%) patients in the EVT group underwent stent-graft treatment of the eroded vessels and 28 (78%) coil embolization. Six (9%) patients underwent reintervention with no significant differences between EVT and OS groups (11% vs. 7%, *p* = 0.692). Postoperative morbidity and complications in 52% of all patients were higher in the OS group than in the EVT group (41% vs. 68%, *p* = 0.029). The in-hospital 30-days mortality rate for all patients was 25%, and it was higher in the OS group than in the EVT group (14% vs. 39%, *p* = 0.017). Conclusions: An endovascular-first strategy for treating visceral arteries erosions may be preferred to reduce the complications associated with open surgery if patients are hemodynamically stable and have no anastomotic insufficiency. Endovascular treatment may be associated with better in-hospital survival when compared to primary open surgery. Further studies are required to identify the optimal approach.

## 1. Introduction

Visceral artery erosion is a rare but life-threatening condition following pancreatic surgery, pancreatitis, or upper gastrointestinal tract malignancies [1,2]. Whereas hemorrhagic complications after pancreatitis are rare, occurring in 1.3% of pancreatitis cases [3], these complications are common after pancreatic surgery, accounting for 5–16% of post-pancreatectomy complications and 1–38% of overall mortality [4,5].

Autodigestion of pancreatic tissue by enzymatic injury was widely accepted as a cause of acute pancreatitis and the development of bleeding [3,6]. However, this hypothesis has been recently reviewed. Other hypotheses included microvascular injury induced by pancreatic ischemia-reperfusion or intravascular thrombosis, which activate the release of active inflammatory peptides [6,7,8]. In addition, pancreatic leakage and infections are frequent complications after pancreatic surgery. Surgical techniques that clear arteries of the surrounding tissue, such as lymphadenectomy, may expose the blood vessel directly to pancreatic enzymes or inflammation, which results in a predisposition to arterial vessel erosion [9].

Treatment options are either an open surgical approach or endovascular interventions, including endovascular embolization or implantation of covered stent-grafts [10,11]. While descriptions of perioperative complications following pancreatic resection can be found numerously in the literature [12,13,14,15,16,17,18,19], there is a lack of literature that reports on and compares the outcome of open surgical and endovascular treatments of the hemorrhagic complications [20].

This study, therefore, aims to report our experience in treating hemorrhagic complications of the visceral arteries and compare the outcomes of different treatment options.

## 2. Materials and Methods

This study was conducted in the Department of Vascular Surgery, Department of Surgery, and Department of Radiology at Charité—Universitätsmedizin Berlin, Germany

During the investigated period, a total of 1645 pancreatectomy procedures were performed in our hospital. However, hemorrhagic complications of the visceral arteries were also found after other pathologies, including malignancies of the upper gastrointestinal tract and pancreatitis. Therefore, a retrospective review of the diagnostic database was performed to identify patients with a rupture of an artery, including arterial erosions and fistulas (International Classification of Disease (ICD-10-GM) code I77.2) who were treated at our hospital between January 2011 and December 2020. After reviewing the medical records of all patients, only patients with visceral arterial erosion (VAE) were enrolled in the study.

A total of 381 patients were identified with the ICD-10-GM code I77.2 in the study period. Of them, 65 patients presented with extraluminal hemorrhage due to visceral artery erosions.

We collected data on patient demographics (gender, age), cardiovascular risk factors, preexisting medical conditions, malignancies, previous cancer surgeries, clinical presentation, and imaging. Risk factors were recorded using the ASA Physical Status Classification System. Diagnosis and detection of the eroded vessels were obtained by computed tomography (CT) or angiography.

We divided the patients according to the etiology of the bleeding into two groups: tumor-related visceral arterial erosion (TR-VAE) and non-tumor-related visceral arterial erosion (NTR-VAE). TR-VAE includes all patients who had bleeding due to the malignancies of the upper abdomen or its surgical treatment. Additionally, NTR-VAE includes all patients who developed spontaneous bleeding of the visceral arteries or as a result of pancreatitis without evidence of malignancies.

Additionally, we divided the patients according to their primary treatment of the visceral arteries into endovascular treatment (EVT) and open surgery (OS) groups. The primary success of the treatment was defined according to its ability to stop the bleeding and avoid reintervention. Reintervention was defined as the need for a new procedure to stop the bleeding after failed primary treatment or recurrent bleeding. The treatment options were discussed in a multidisciplinary session, including surgeons and radiologists. Endovascular treatment was preferably used whenever it was possible. In the cases of abscess, anastomosis insufficiency, or hemodynamic instability, a primary open surgical treatment was conducted.

According to the endovascular treatment, we used femoral or brachial artery access. The eroded vessels and the source of bleeding were identified using the arteriogram and then treated using stent-grafts and/or coils or particle embolization. Primary open surgical treatment of the visceral arteries was preferred if the open surgery was indicated for other reasons like an abscess or anastomosis insufficiency. Additionally, patients with failed endovascular treatment were treated with open surgery.

Procedural data included technical details of endovascular and open surgical procedures, primary or secondary, number of coils, and stents.

Endpoints included in-hospital and one-year mortalities and postoperative complications.

### Statistical Analysis

Categorical data are reported as frequencies (percentages) and continuous variables as median (range). Comparisons of continuous data were achieved by the Mann–Whitney U-test and categorical data were compared using the chi-squared test and Fisher’s exact test. Survival curves were analyzed using Kaplan–Meier. Differences in survival between the groups were analyzed using the log-rank test. Values with a confidence interval of 95% corresponding to *p* ≤ 0.05 were considered statistically significant. All analyses were performed using SPSS 25 (IBM SPSS Statistics version 25, IBM Deutschland GmbH).

## 3. Results

### 3.1. Patient Cohort

A total of 381 patients were identified with the ICD-10-GM code I77.2 (rupture of artery including arterial erosions and fistulas) between 2011 and 2020. Of them, 65 patients (46 males, mean age 63 ± 14 years) presented with extraluminal hemorrhage due to visceral artery erosions. Demographics, risk factors, and etiology are depicted in Table 1.

A total of 49 (75%) were TR-VAEs and 16 (25%) were NTR-VAEs. The malignancies of the patients in the TR-VAE group included pancreatic cancers in 31 (59%) patients, liver and bile duct cancers in 11 (21%) patients, and stomach and duodenal cancers in 7 (13%).

Four (8%) patients in the NTR-VAE group had malignancies in other locations; however, the bleeding was not related to the tumor as described in Table 2.

The causes of erosion in all patients included complications of surgical treatment for the pancreas and upper gastrointestinal tract (*n* = 49 patients, 75%), pancreatitis (*n* = 12 patients, 19%), and spontaneous bleeding with a tumor in other locations (*n* = 4 patients, 6%).

Pancreatectomy was obtained in 34 (52%) patients in the current study, representing 2% of all pancreatectomy procedures (*n* = 1645) performed in our hospital during the study period. The pancreatectomy procedures included pylorus preserving pancreatoduodectomy (PPPD) in 22 patients, Whipple procedure in 4 patients, and distal pancreatic resection in 8 patients.

Other surgical treatments included hepatectomy in 6 (9%) patients, bile duct resections in 3 (5%), gastrectomy in 6 (9%), and splenectomy in 12 (19%) patients. The median time between the previous procedure and the erosion of the visceral artery was 14 days (range, 2–61 days).

According to the eroded vessels, the celiac artery and its branches were the most commonly eroded vessels (*n* = 60, 92%), followed by the superior mesenteric artery (*n* = 3, 5%) and the inferior phrenic artery (*n* = 2, 3%) see Table 3.

### 3.2. Treatment Options for Erosion Bleeding

The bleeding source could be localized in 37 of 41 (90%) patients who underwent angiography and 21 of 36 (58%) patients who underwent CT-angiography. As a result, in 37 (57%) patients, endovascular treatment (EVT) and in 28 (43%) patients open surgery (OS) was applied as primary treatment. According to the primary treatment options, there was no statistically significant difference between tumor-related and non-tumor-related bleeding. The decision to make primary open surgery was because of hemodynamic instability in eight (29%) patients and the need for open surgical intervention for other reasons in 20 (71%), including anastomotic insufficiency in 14 (50%) patients or pancreatic fistula in six (21%) patients. Surgical procedures included simple ligation of the bleeding arteries in 14 (50%) patients, oversewing the bleeding site in eight (29%) patients, vascular reconstruction in two (7%), and splenectomy in four (14%) patients. Forty-one (63%) patients needed blood transfusion (median 5 PRBCs, range 2–42 PRBCs) due to the erosion bleeding with no significant difference between EVT and OS groups (*p* = 0.915).

**Figure 1:**
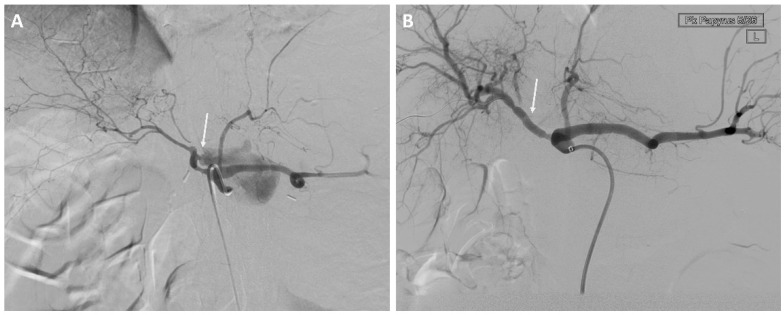
A 61-year-old man with a history of pancreatic head adenocarcinoma and pylorus preserving pancreatoduodectomy (PPPD). (**A**) Angiography showing bleeding from the gastroduodenal artery stump and the hepatic artery. (**B**) Treatment of the hepatic artery with a stent-graft.

**Figure 2:**
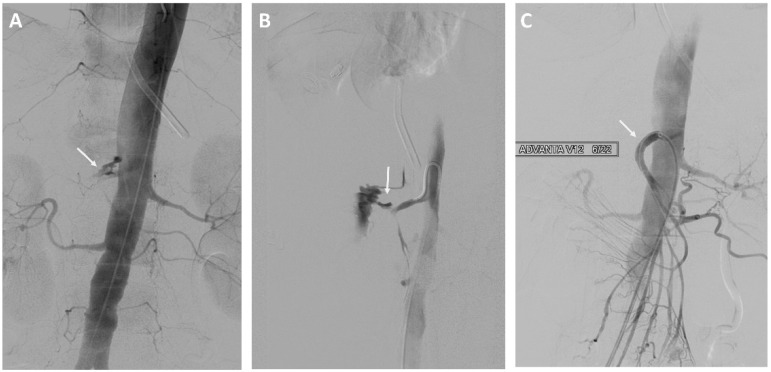
A 73-year-old woman with a history of intraductal papillary mucinous cancer of the pancreatic head and pylorus preserving pancreatoduodectomy (PPPD). (**A**,**B**) Angiography showing bleeding from the superior mesenteric artery. (**C**) Treatment of the superior mesenteric artery with a stent-graft.

Eight of 37 (22%) patients in the EVT group underwent stent-graft treatment of the eroded vessels: celiac artery (*n* = 1), common hepatic artery (*n* = 4) see Figure 1, right hepatic artery (*n* = 1), and superior mesenteric artery (*n* = 2) see Figure 2. The stent-grafts used were Advanta V12TM (Getinge, Gothenburg, Sweden) in five patients, PK Papyrus covered stent (BIOTRONIK, Bulach, Switzerland) in one, covered balloon-expandable Jomed stent (Abbott Vascular, Santa Clara, CA, USA) in one, and polytetrafluoroethylene (PTFE)-covered Jostent GraftMaster^®^ (Abbott Vascular, Santa Clara, CA, USA) in one. Technical success was achieved in all cases.

**Figure 3:**
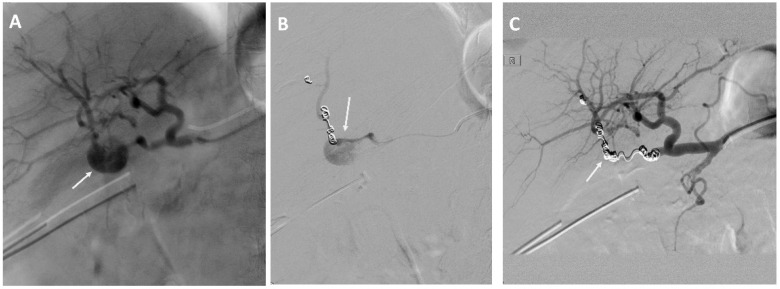
A 74-year-old man with a history of cholangiocarcinoma of the extrahepatic bile ducts treated with resection of the bile ducts and biliodigestive anastomosis. (**A**) Angiography showing bleeding from the right hepatic artery. (**B**,**C**) Endovascular coil embolization of the right hepatic artery.

Coiling of the eroded vessels was done in 28 (78%) of the EVT patients and in three (10%) of the OS patients who developed recurrent bleeding. The median number of coils used was seven coils (range, 2–23 coils) see Figure 3.

A total of six (9%) patients underwent reintervention or redo operation due to recurrent bleeding with no significant differences between primary endovascular and open surgical treatment (11% vs. 7%, *p* = 0.692).

Therefore, freedom from reintervention was 89% after primary endovascular treatment and 93% after primary open surgical treatment. Recurrent bleeding was the cause of reintervention in all patients. Two patients who underwent primary open surgery for bleeding of the common hepatic artery had had recurrent bleeding after vessel suture and needed endovascular coiling of the common hepatic artery and its branches to stop the bleeding. One patient recompensated after the occlusion of the common hepatic artery, and the other developed acute ischemic hepatitis and died five days later.

### 3.3. Outcomes

**Figure 4:**
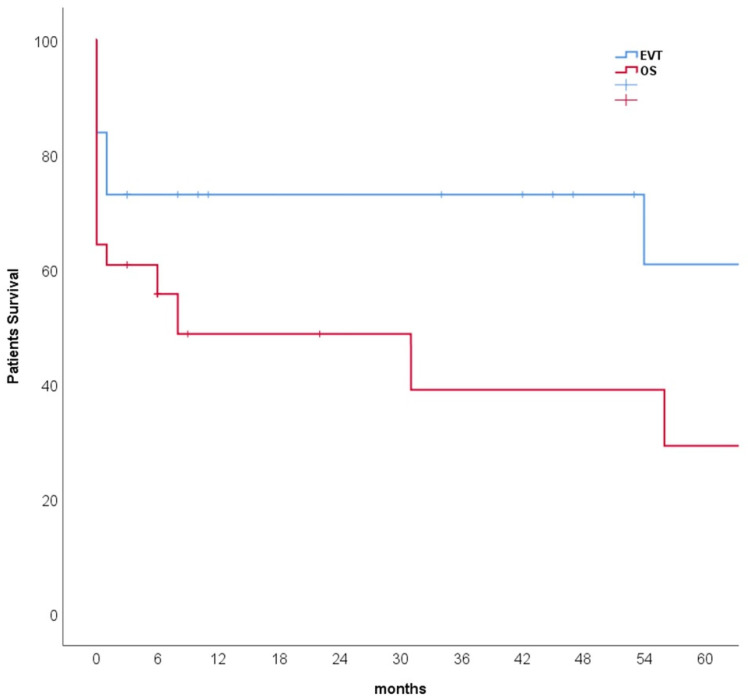
Kaplan–Meier survival estimates of endovascular treatment (EVT) and open surgery (OS) of the bleedings after visceral artery erosions; Kaplan–Meier log-rank test, *p* = 0.061.

The median follow-up for the tumor-related patients was three months (range, 0–128 months), and for the non-tumor-related bleeding was 20 months (range, 3–98). Postoperative morbidity and complications in 52% of all patients were higher in the OS group than in the EVT group (41% vs. 68%, *p* = 0.029). The in-hospital 30-day mortality rate for all patients was 25% (*n* = 16), and it was higher in the OS group compared to EVT group (14% vs. 39%, *p* = 0.017). However, the one-year mortality rate was 35% (*n* = 23) with no statistically significant difference between EVT and OS groups (27% vs. 46%, *p* = 0.105). A Kaplan–Meier analysis contrasting survival between groups is shown in Figure 4. In detail, 26 (40%) patients died during the follow-up. The causes of deaths included acute hemodynamic collapse directly on the operating table (*n* = 2), hemorrhagic shock in the intensive care unit (*n* = 5), multiple organ dysfunction syndromes (MODS) despite initial successful hemostasis (*n* = 14), and cancer-related deaths (*n* = 5).

## 4. Discussion

In the current study, we found that malignancies of the upper abdomen and gastrointestinal tract and their surgical treatment were the most common cause for the erosion of the visceral arteries, while pancreatitis was the most common cause in non-malignant patients. Additionally, the in-hospital 30-day mortality of the patients treated with endovascular options was statistically lower compared to patients treated with open surgery.

Despite the low number of cases, many studies showed that endovascular options are effective and safe for treating delayed visceral arterial hemorrhage following a pancreatectomy procedure [21,22,23]. Similar results were found in our study with a primary technical success in stopping the bleeding of 89% and lower in-hospital mortality compared to the open surgical options.

Many hypotheses have been discussed about the etiology of the erosion of the visceral arteries. The destruction of the elastase in the arterial wall by proteolytic enzymes or direct erosion of the vessel by a pseudocyst, pancreatic necrosis, or abscess may be the most common mechanisms of the bleeding [6,7]. Additionally, many factors such as local tissue injury and systemic inflammatory responses might be involved in microvascular injury in acute necrotizing pancreatitis leading to erosion bleeding [24,25,26]. Although microvascular pathomechanisms seem to be rather understood, causes for macrovascular complications such as erosion bleeding are not fully understood. However, the involvement of vasa vasorum of macrovessels is discussed as a plausible cause [6]. In our study, the presumed causes of the vessel erosions include pancreatic secret and local infections.

In general, there are two different strategies for treating the eroded vessels: Open surgical and endovascular approaches. Our study suggests that a primary endovascular approach is the first therapeutic option whenever possible. However, a primary open surgical procedure may be mandatory if the bleeding is accompanied by anastomosis insufficiency, pancreatic fistula, or hemodynamic instability. Some studies reported a high technical success rate of stent-graft placement [21,27]. Additionally, choosing between stenting and coiling of the visceral arteries is dependent on the location and the size of the eroded vessels. For example, a stent-graft has to be considered if the erosion occurs in the main celiac or superior mesenteric artery because of the possible consequences of liver or bowel ischemia after occlusion of the main artery [6,22,23,27]. On the other hand, endovascular coiling or embolization may be sufficient if the erosion occurs in the branches of the main arteries [28,29,30,31]. However, recurrent bleeding can occur in up to 21% of the patient after endovascular management [32]. In our study, 11% of the patients developed recurrent bleeding after endovascular treatment.

In the presence of an anastomosis insufficiency, covered stent-grafts might result in graft infection when contact with the gastrointestinal content occurs [21,33]. A graft infection was described in none of the patients included in our analyses during follow-up.

A management algorithm of the post-pancreatoduodenectomy hemorrhage was proposed and discussed in many studies [34,35,36,37]. The post-pancreatectomy hemorrhage patients were graded according to the time of onset (early ≤24 h, late >24 h), location (intraluminal, extraluminal), and severity of hemorrhage (mild, severe). There were no bleedings in the first 24 h after surgery, and all patients had extraluminal bleedings in our study.

Also, our study suggests that indications for both treatment options—endovascular treatment or open surgery—have to be considered thoroughly; a primary open surgical approach might be the better treatment option if anastomosis insufficiencies or pancreatic fistulas complicated the cases.

Müssle et al. reported reduced incidence of erosion hemorrhage by using the falciform ligament wrap at the initial pancreatoduodenectomy to cover the gastroduodenal artery stump [38,39]. However, none of the patients in our study received a falciform ligament wrap.

Any of the pancreatic and peripancreatic arteries may be involved in erosion bleeding. However, some studies found that splenic or gastroduodenal arteries were the most frequent origin of the post-pancreatectomy hemorrhage [6,40]. In our study, the common hepatic artery was the most common source of bleeding, followed by the splenic artery and gastroduodenal artery.

Our study showed that the 30-day mortality rate was significantly lower in the endovascular treatment group. However, at one-year follow-up, no statistical significance between the two groups was observed anymore, indicating that mid-term and long-term survivals are related to the staging and prognosis of the primary tumor. Therefore, the aim of the endovascular and open surgical treatment is not to change the prognosis of the patients but to stop the hemorrhage in emergencies.

This study is limited by its retrospective nature and the relatively small number of patients due to the rarity of this disease. Additionally, there is some selection bias between endovascular and open surgical treatment because most patients who needed open surgery had had other severe complications, which make the prognosis of these patients worse.

## 5. Conclusions

Visceral arterial erosion bleeding remains a complication with high mortality rates. The endovascular-first strategy seems to be reliable in controlling emergency bleeding. However, open surgery has to be performed if the patients develop other complications of the initial surgery. Endovascular implantation of stent-grafts or coiling of the bleeding artery is associated with better in-hospital survival than primary open surgery.

## Figures and Tables

**Table 1:** Descriptive characteristics and etiology.

Characteristic	Total *n* (%)	EVT *n* (%)	OS *n* (%)	*p*-Value
Patients	65	37 (57)	28 (43)	
Age	62 ± 14	63 ± 13	62 ± 15	0.678
Male	46 (71)	25 (67)	21 (75)	0.514
Risk factors				
Coronary artery disease	5 (8)	4 (11)	1 (4)	0.380
Diabetes mellitus	16 (25)	9 (24)	7 (25)	0.950
Hypertension	26 (40)	16 (43)	10 (38)	0.540
COPD	4 (6)	3 (8)	1 (4)	0.628
Smoking	6 (9)	4 (11)	2 (7)	0.692
Chronic renal insufficiency	7 (11)	2 (5)	5 (18)	0.224
ASA classification ^1^				
I	1 (2)	0 (0)	1 (5)	
II	21 (44)	9 (35)	12 (55)	
III	25 (52)	16 (62)	9 (41)	
IV	1 (2)	1 (4)	0	
Etiology				
Complications of pancreatic surgeries	49 (75)	24 (65)	25 (89)	0.024 *
Pancreatitis	12 (19)	9 (24)	3 (11)	0.161
Spontaneous bleeding	4 (6)	4 (11)	0	

^1^ ASA: American Society of Anesthesiologists; EVT: endovascular treatment; OS: open surgery; * *p*-Value < 0.05.

**Table 2:** Malignancies, surgical procedures, and surgical complications. All percentages are calculated from the whole patient’s cohort *n* = 65.

Malignancy	Total *n* (%)
Pancreatic cancer	31 (48)
Head	22 (34)
body	4 (6)
tail	3 (5)
^1^ IPMN	2 (3)
Liver & bile duct	11 (17)
Gallbladder cancer	1 (2)
Klatskin tumor	2 (3)
Cholangiocarcinoma	6 (9)
Hepatocellular carcinoma	2 (3)
Stomach and duodenum	5 (8)
^2^ OGJA	2 (3)
^3^ DLBCL	1 (2)
^4^ SRCC	1 (2)
Duodenal cancer	1 (2)
Others	4 (6)
Uterine cancer	1 (2)
Breast cancer	1 (2)
Bladder cancer	2 (3)
Previous surgical procedures	49 (75)
Pancreatectomy	34 (52)
Hepatectomy	6 (9)
Bile duct resection	3 (5)
Gastrectomy	6 (9)
Splenectomy	12 (19)
Previous complications	
Anastomotic insufficiency	21 (32)
Pancreatic fistula	11 (17)
Pancreatic cyst	3 (5)

^1^ IPMN: intraductal papillary mucinous neoplasm; ^2^ OGJA: esophagogastric junction adenocarcinoma; ^3^ DLBCL: diffuse large B-cell lymphoma; ^4^ SRCC: signet ring cell carcinoma.

**Table 3:** Eroded vessels according to the treatment options.

Eroded Vessels	Total 65 (100)	^1^ EVT 37 (57)	^2^ OS 28 (43)
**Branches of the celiac artery**	60 (92)	34 (92)	26 (93)
Celiac trunk	2 (3)	0	2 (7)
Splenic artery	13 (20)	5 (14)	8 (29)
Common hepatic artery	18 (28)	9 (24)	9 (32)
The left branch of the hepatic artery proper	1 (2)	1 (3)	0
The right branch of the hepatic artery proper	9 (14)	7 (19)	2 (7)
Left gastric artery	3 (5)	2 (5)	1 (4)
Light gastric artery	3 (5)	2 (5)	1 (4)
Gastroduodenal artery	10 (15)	8 (22)	2 (7)
**Superior mesenteric artery**	3 (5)	3 (8)	0
**Inferior phrenic artery**	2 (3)	0	2 (7)

Data are presented as *n* (%); ^1^ EVT: endovascular treatment; ^2^ OS: open surgery.

## Data Availability

The data presented in this study are available on request from the corresponding author.
